# Anatomical Variations That Can Lead to Spine Surgery at the Wrong Level: Part III Lumbosacral Spine

**DOI:** 10.7759/cureus.9433

**Published:** 2020-07-28

**Authors:** Manan Shah, Dia R Halalmeh, Aubin Sandio, R. Shane Tubbs, Marc D Moisi

**Affiliations:** 1 Neurosurgery, Wayne State University/Detroit Medical Center, Detroit, USA; 2 Neurosurgery and Structural & Cellular Biology, Tulane University School of Medicine, New Orleans, USA; 3 Anatomical Sciences, St. George's University, St. George's, GRD; 4 Neurosurgery and Ochsner Neuroscience Institute, Ochsner Health System, New Orleans, USA

**Keywords:** surgery at the wrong level, lumbosacral spine, anatomical variations, spine, sentinel events, lumbar, sacral

## Abstract

Spine surgery at the wrong level is an undesirable event and unique pitfall in spine surgery. It is detrimental to the relationship between the patient and the surgeon and typically results in profound medical and legal consequences. It falls under the wrong-site surgery sentinel events reporting system. This error is most frequently observed in lumbosacral spine. Several risk factors are implicated; however, anatomical variations of the lumbosacral spine are a major risk factor. The aim of this article was to provide a detailed description of these high-risk anatomical variations, including transitional vertebrae, lumbar ribs, butterfly vertebrae, hemivertebra, block/fused vertebrae, and spinal dysraphism. A literature review was performed in the database PubMed to obtain all relative English-only articles concerning these anatomical variations and their implication in the development of lumbosacral spine surgery at the wrong level. We also described patient characteristics that can lead to lumbosacral surgery at the wrong level such as tumors, infection, previous lumbosacral surgery, obesity, and osteoporosis. Certain techniques to prevent such incorrect surgery were explained. Lumbosacral spine anatomical variations are surgically significant. Awareness of their existence may provide better pre-operative planning and surgical intervention, leading to avoidance of incorrect-level surgery and potentially better clinical outcomes. In addition, collaboration with radiologists and careful examination of patient’s anatomy and characteristics should be exercised, especially in difficult cases.

## Introduction and background

Spine surgery at the wrong level is one of the most detrimental surgical errors that a surgeon can make. It can lead to additional procedures and risks, damage the doctor-patient relationship, and result in legal actions [1]. The term “wrong-site surgery” was created as a concept that includes such actions as operating on the wrong person, organ or limb, or vertebral level [1,2]. In 2008, the Joint Commission on Accreditation of Healthcare Organization (JCAHO) reported that wrong-site surgery was the most common sentinel event (13%) [1]. The incidence of surgery at the wrong level reported in the literature ranges from 0.09 to 4.5 per 10,000 surgeries performed. One survey estimated that half of spine surgeons will perform at least one procedure at the wrong level during their careers [3]. Such surgical errors are more prevalent in the lumbosacral spine than in the cervical or thoracic spine [3]. Potential risk factors include emergency surgery, unusual time pressure to begin or complete the procedure, involvement of multiple surgeons or multiple procedures in a single surgical visit, failure to verify operative site because of suboptimal radiographs, failure to recognize aberrant anatomy, unusual patient characteristics, vertebral miscounting, failure to re-localize after exposure, and lack of communication [1,4,5]. These unusual patient characteristics and aberrant anatomy in the lumbosacral spine are important to identify and analyze in order to prevent surgery at the wrong level.

In this article, we present several lumbosacral spine anatomical variations that can potentially lead to surgery at the wrong level, including transitional vertebrae, lumbar ribs, butterfly vertebrae, hemivertebrae, block/fused vertebrae, and spinal dysraphism. We also explain the way certain characteristics, such as tumors, infection, previous lumbosacral surgery, obesity, and osteoporosis, can lead to surgery at the wrong level. Moreover, we demonstrated certain techniques that can help prevent this devastating event.

## Review

Materials and methods

The study was a thorough literature review of the English-only journals using the PubMed database. The search terms included “surgery at the wrong level,” “lumbosacral transitional vertebra,” “butterfly vertebra,” “lumbar rib,” “lumbar hemivertebra,” “lumbar spinal dysraphism,” “lumbosacral anomalies,” “obesity and spine surgery,” and “osteoporosis and spine surgery.” The search was performed over a period of three months, between October 2018 and December 2018, for relevant studies from the last 38 years, and the references of all primary studies were inspected for additional references not identified in the initial search. Articles exclusively related to lumbosacral anatomical variations and their role in surgery at the wrong level were included. In addition, publications on unusual patient characteristics that can potentially lead to this pitfall were also included. After removal of articles on the basis of non-English language literature, unavailability of full texts, and duplications, the remaining articles were screened for the required information.

Results and discussion

The literature searches revealed over 8,000 references using the initial search terms noted in the methods section. After filtering the articles and further review, 37 peer-reviewed articles were used in this literature review to discuss spine surgery at the wrong level and the lumbosacral anomalies that can cause it. The studies included were published from 1981 to 2018. The results comprised potential risk factors for lumbosacral spine surgery at the wrong level, importantly anatomical variations, and they were discussed as follows:

Transitional Vertebrae

**Figure 1:**
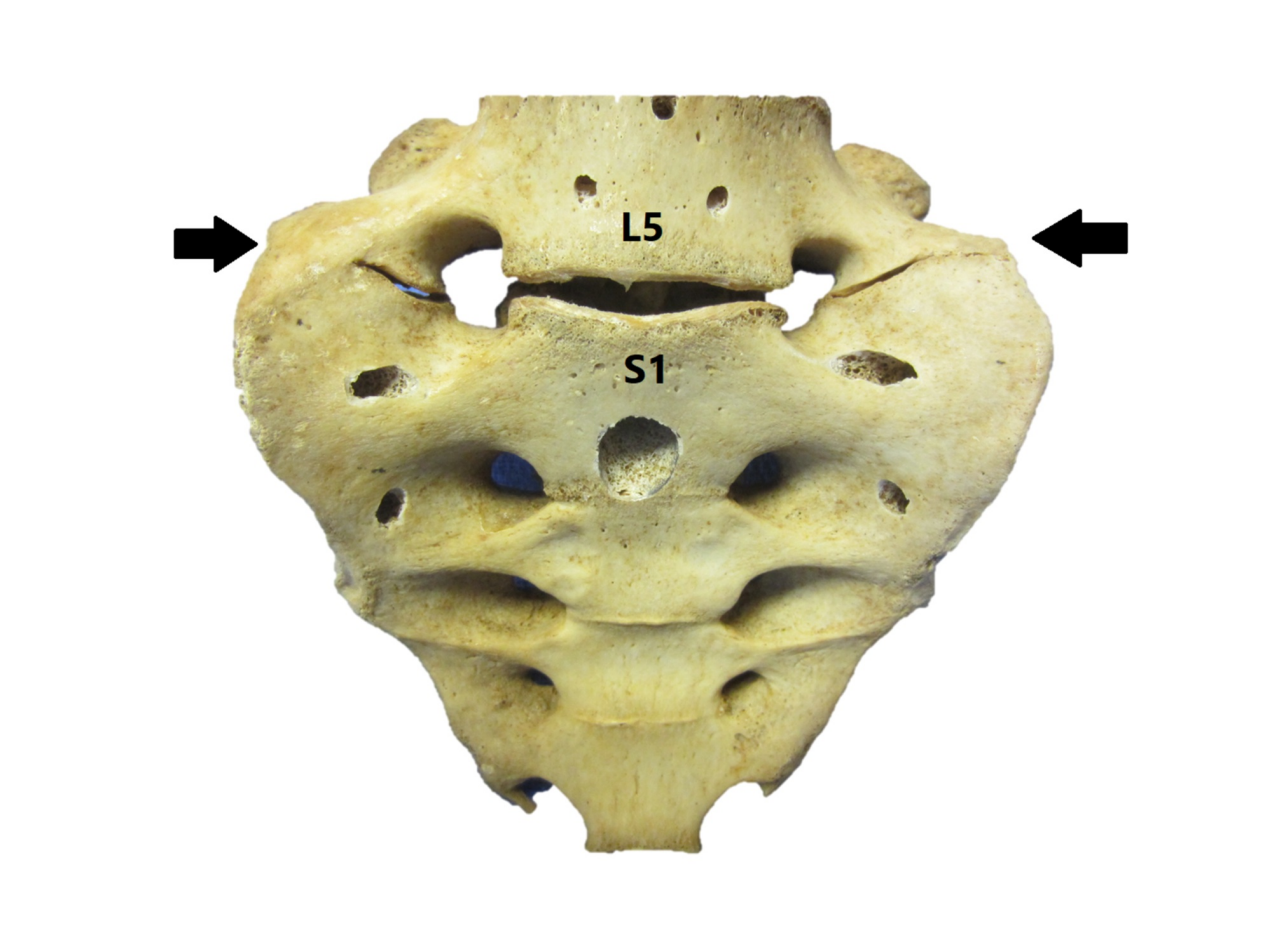
Sacralization of the fifth lumbar vertebrae Specimen showing complete sacralization of the fifth lumbar vertebrae (arrows). The sacralized vertebra now represents the first sacral vertebra. This phenomenon is recognized in the general population with a prevalence of 4%-30% [6-9]

Similar to thoracolumbar transitional vertebrae, lumbosacral transitional vertebrae (LSTV) are common spinal anomalies that potentially can lead to surgery at the wrong level [6]. These are defined either as sacralization of the spine’s lowest lumbar segment or lumbarization of the most superior sacral segment (Figure 1) [7-9]. The prevalence of these vertebrae is approximately 4%-30% according to the literature [7-10]. Formerly, they were best visualized on Ferguson radiographs, which are AP radiographs angled cranially at 300, but currently, CT is the imaging method of choice [8]. MR imaging is used typically for surgical cases, but this is problematic, as it is highly difficult to classify and number LSTV on MRI [8].

In 1984, Castellvi et al. created a classification system for the types of LSTV based on morphology [11]. Type I includes unilateral (Ia) or bilateral (Ib) dysplastic transverse processes that are at least 19 mm wide (craniocaudal dimension); type II includes incomplete unilateral (IIa) or bilateral (IIb) lumbarization/sacralization with an enlarged transverse process forming a diarthrodial joint with lateral mass of the sacrum; type III includes unilateral (IIIa) or bilateral (IIIb) lumbarization/sacralization with complete osseous fusion of the transverse process(es) to the sacrum, and type IV involves a unilateral type II transition with a type III on the contralateral side [8]. Retrospectively, Apazidis et al. reviewed a total of 1,100 abdominal films, and identified 211 eligible films to establish the prevalence rate for LSTV in the American general population [7]. They found that 75 (35.6%) of the subjects had an LSTV and the most frequent anatomical variant was Castellvi et al.’s type IA (14.7%).

Accurate numerical identification of the vertebra is essential to prevent surgery at the wrong level. Radiographs of the entire spine allow radiologists to count from C2 inferiorly, differentiate hypoplastic ribs from lumbar transverse processes, and identify the L1 vertebral body correctly, facilitating the LSTV’s correct numerical identification [8]. Wigh reviewed operative reports and myelogram findings of 42 patients with transitional vertebrae and found five cases of surgery at the wrong level attributable to nomenclature error [12]. Hahn et al. were the first to describe using a sagittal cervicothoracic MR localizer to evaluate transitional vertebrae better, as in this way, the vertebrae can be counted caudad from C2 rather than cephalad from L5 [13]. The addition of a coronal MR cervicothoracic localizer also increases the accuracy with which these anomalies can be labeled numerically [8,9,14]. Another method that also has been suggested is identifying the right renal artery on a T1-weighted paramedian sagittal MRI because this artery usually lies at or near the L1-2 disc [8,9,15]. Ultimately, without high-quality imaging of the entire spine, there is no definite method to number a transitional segment accurately. Accordingly, the surgeon must review preoperative and intraoperative imaging carefully and be consistent when numbering the LSTV to avoid operating at the wrong level.

Lumbar Ribs

**Figure 2:**
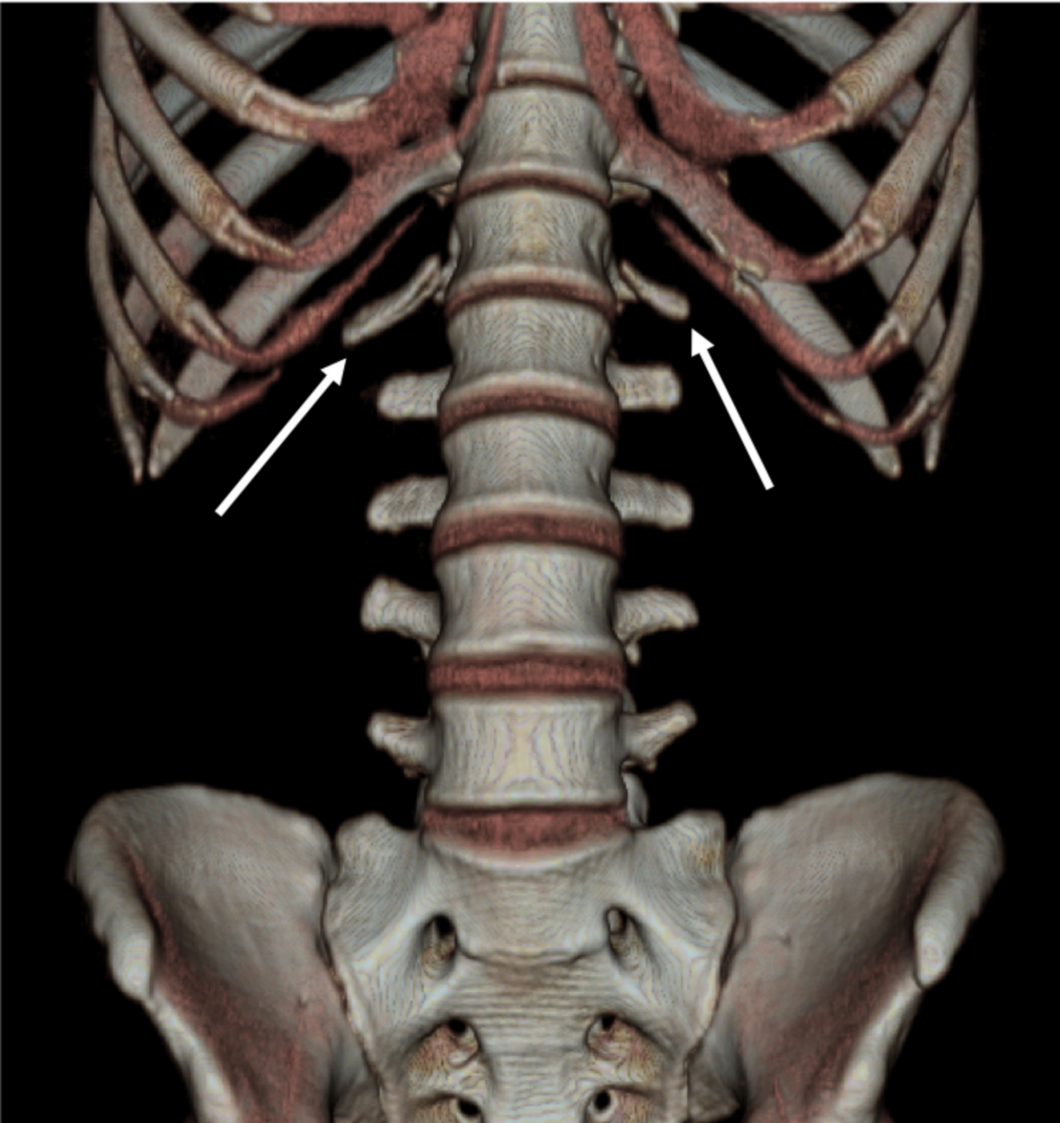
Lumbar ribs 3D computed tomography showing lumbar ribs (arrows) at L1. They are noticeably shorter and more horizontal compared to the thoracic ribs [15]

A relatively uncommon anomaly of the lumbar region is the outgrowth of a lumbar rib, which is a rudimentary or “extra” rib that occurs in approximately 1% of the population. These ribs appear to be floating and have an appearance similar to normal ribs but follow a different course. Lumbar ribs are differentiated from thoracic ribs by their length, which is half, or less than half, of the adjacent thoracic rib, and their course, which is more horizontal and tapers upward at the distal aspect (Figure 2) [16]. Aly et al. demonstrated that lumbar ribs may be bilateral or unilateral on L1 [16]. Most are unilateral, particularly on the right, and are mistaken for osteophytes, transverse process anomalies, or abdominal vessel anomalies [16]. Mahajan et al. suggested that lumbar ribs result from incomplete fusion of the sclerotome’s cranial and rostral segments during embryological development [17]. To diagnose these accessory ribs, CT imaging can be used to identify the associated costovertebral joint, as simple elongated transverse processes of lumbar vertebrae do not have such a joint [16]. Nakajima et al. found 13 cases of lumbar ribs in 288 cases of lumbarization [18]. In their series, 78.6% of cases of lumbarization were associated with lumbar ribs and 84.6% of cases of lumbar ribs were associated with lumbarization, in which the counting error rate for the lumbar spine was approximately 10% in these cases [18]. For cases with lumbar ribs, reviewing CT imaging is an ideal way for surgeons to determine the proper level when operating.

Butterfly Vertebra

**Figure 3:**
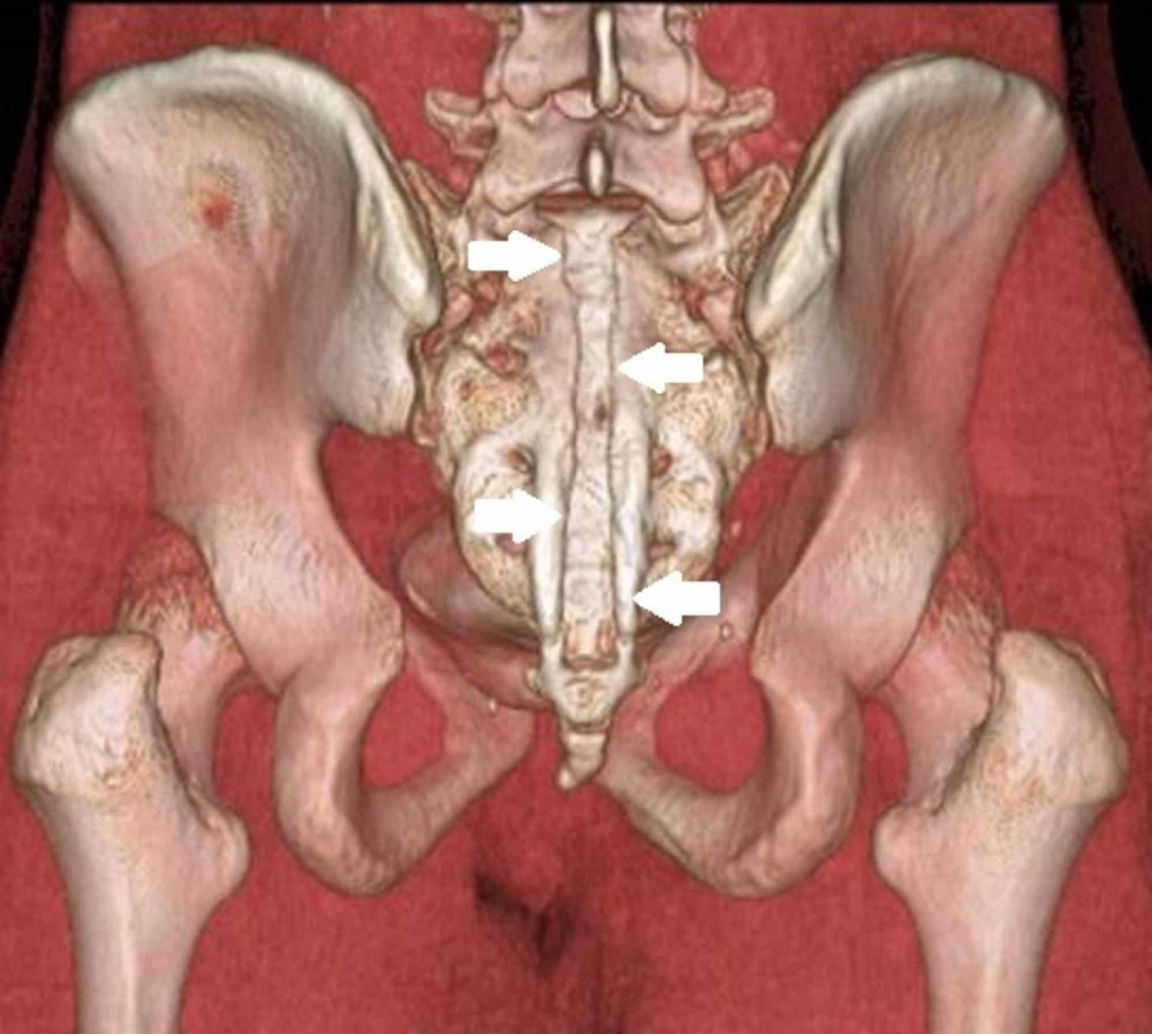
Butterfly vertebrae Butterfly vertebra, also known as, spina bifida, of the sacrum. The defect is characterized by anterior and median aplasia [20,21]. The sacral vertebra has a prominent midline cleft (arrows) through the body, giving the sacrum a funnel shape at the caudal end, hence the butterfly appearance on imaging

Butterfly vertebra is another rare congenital anomaly that can be found in the lumbar spine (Figure 3). It also is referred to as cleft vertebra, sagittal cleft vertebra, and anterior rachischisis, somatoschisis, and spina bifida [19,20].

It represents a defect in the vertebral body formation characterized by anterior and median aplasia [21,22]. The vertebra has a cleft through the body and a funnel shape at the ends, which gives it a butterfly appearance on AP radiographs. Typically, it occurs in the lumbar spine and can be an isolated finding or can be associated with other congenital syndromes, such as Alagille’s, Jarcho-Levin’s, and Pfeiffer’s syndromes [20,21]. The body’s butterfly shape can be seen easily on simple AP radiographs, while the pedicles may look divergent. However, in lateral radiographs, the butterfly vertebra appears wedge-shaped and thus can be confused with a compression fracture. There also are degenerative changes of the intervertebral discs above and below the butterfly vertebra that can make it more complicated to identify lumbar vertebra on intraoperative x-rays [21].

Hemivertebra

**Figure 4:**
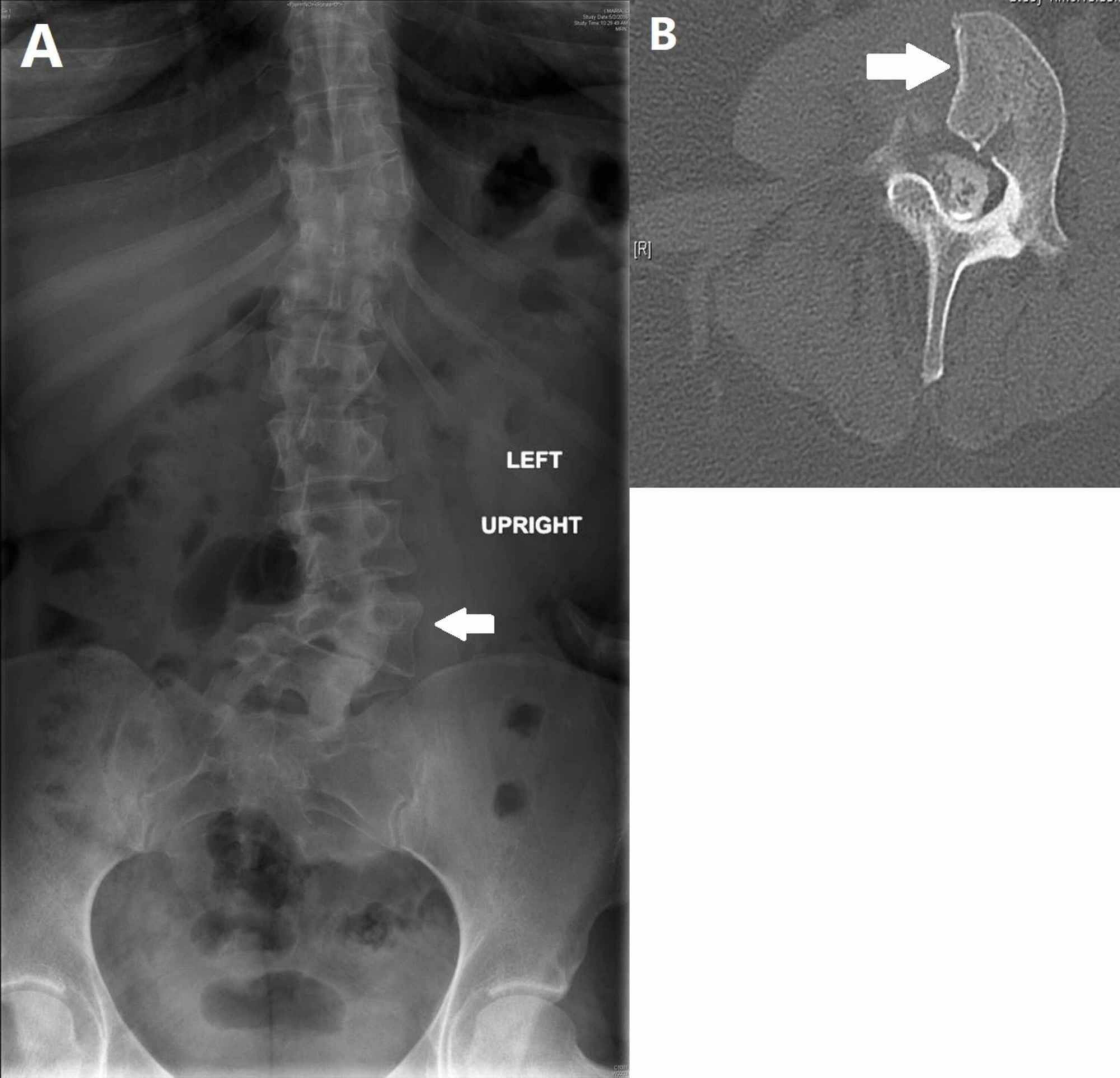
Lumbar hemivertebrae Anterior-posterior radiograph showing a lumbar hemivertebrae (arrow) and associated spinal curvature (A). An axial view of the same defect (arrow) on MR imaging (B)

Hemivertebra is a spinal congenital anomaly that results from lack of formation of one half of the vertebral body. It occurs in 5-10/10,000 births with a female predominance [23]. It is believed to be due to the failure of chondrification centers to develop during fetal life (Figure 4) [23]. Four types of hemivertebra are described: incarcerated, nonincarcerated, segmented, and unsegmented [24]. Incarcerated hemivertebrae are typically imprisoned within lateral margins of two adjacent vertebrae above and below. Conversely, in nonincarcerated type, the contiguous normal vertebrae are completely separated from each other, housing the wedged hemivertebra in between. Segmented hemivertebra is classically situated between two intervertebral discs, and is associated with more deterioration and subsequent spinal curvature than unsegmented hemivertebra, which has fibrous tissue above and beneath it (i.e., fused with adjacent vertebra) [24]. A study of lumbar spine radiographs found that approximately 0.27% of asymptomatic individuals had hemivertebra [23]. It is very common for hemivertebra to be associated with concomitant congenital anomalies. In Bollini et al.’s study of congenital anomalies, hemivertebrae were observed in the lumbar and lumbosacral region in 35% and 29% of cases, respectively [25]. In the lumbar region, medullary anomalies, such as meningocele, syringomyelia, tethered spinal cord, myelocystocele, and lipoma accompanied hemivertebra 13% of the time [23,25]. Hemivertebrae can cause the spine to angle, resulting in abnormal spinal curvatures such as kyphosis, lordosis, and scoliosis. Occasionally, identification of the target lumbar level intraoperatively can be complicated by this type of vertebral anomalies; therefore, care must be taken when using x-rays. As with hemivertebrae in the cervical and thoracic spine, preoperative imaging, particularly CT scan, may be performed to facilitate identification of the proper level [6,26]. Anterior hemivertebrae are associated frequently with a posterior hemilamina located one level cephalad [27]. Coronal images obtained through CT allow the surgeon to trace the relation between the irregular anterior and posterior structures and prevent confusion intraoperatively. However, a hemivertebra can also be a useful landmark after careful identification.

Block/Fused Vertebra

Failure of separation of adjacent vertebrae results in block or fused vertebra. This vertebral anomaly is most frequently seen in the cervical and lumbar spine, and it is thought to be due to failure of segmentation of somites during development [28]. Fusion of two or more adjacent vertebral bodies is mediated through intervertebral joints and discs. The height of a block vertebra is the sum of the heights of the joined bodies and the intervertebral discs between them. A “waist” can be noted radiologically at the level of the intervertebral disc between the fused vertebral segments [29]. In comparison, acquired fused vertebrae are lower in height, and lack this characteristic radiologic finding. The disparity between the disc height in congenital block vertebrae and acquired fused vertebrae can lead to uncertainty about the spinal level. Furthermore, inability to identify a block or fused vertebra may result in an enumeration error that can lead to surgery at the wrong level [6,26]. In addition, this fusion can disturb the normal anatomical angle of the spine, which complicates the approach further [28]. However, if identified correctly, this lumbar spine variation can assist in proper labeling of other levels during surgery.

Spinal Dysraphism

**Figure 5:**
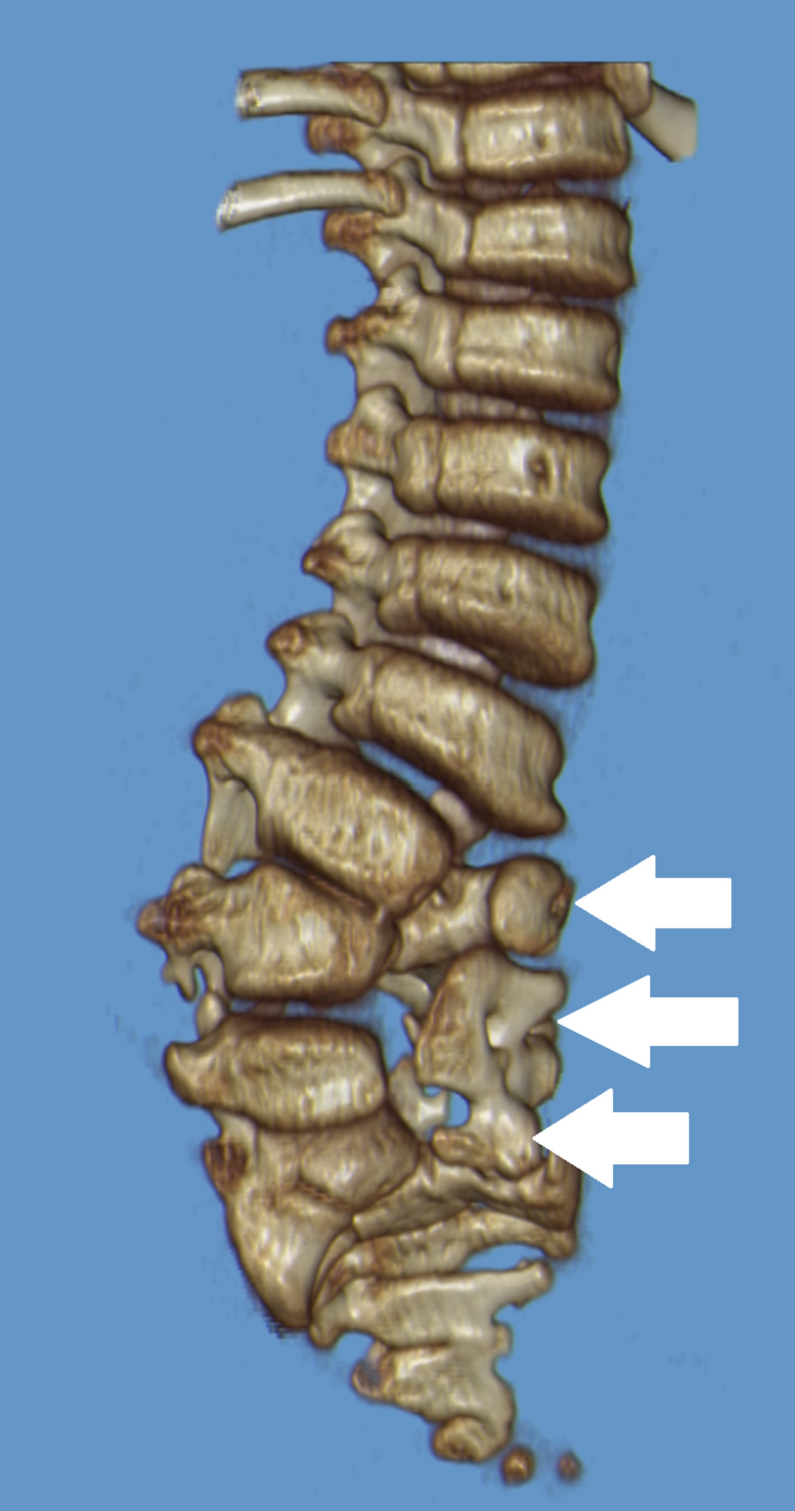
Spinal dysraphism at lumbosacral region Dysraphic lumbosacral junction at multiple levels (arrows) of the lumbar vertebrae and sacrum with subsequent scoliosis

Spinal dysraphism is another congenital anomaly that refers to the neural arch’s failure to fuse, and is associated with central nervous system abnormalities [28]. Spina bifida occulta, which is the closed, mild form, typically involves the spine’s transitional levels, including L5 and S1. The midline defects include an unfused spinous process, laminae, or both (Figure 5). The spinous process may remain unattached, held by ligamentum flavum, or may fuse with the contiguous spinous process, creating the so-called clasp-knife deformity [28,30]. Spinous process deviation can reflect the entire vertebra’s rotation or the neural arch’s developmental asymmetry, which can cause confusion in interpreting AP radiographs [28]. Diastematomyelia is another congenital spine anomaly where part of the spinal cord is split into two hemicords longitudinally. Type 1 is that in which the two hemicords are located within individual dural tubes separated by a cartilaginous or osseous septum, while type 2 is that in which there is a single dural tube that contains two hemicords, sometimes with an intervening fibrous septum [28,31]. These defects in the lumbosacral spine’s posterior elements increase the risk of surgery at the wrong level and must be taken into consideration prior to surgery.

Tumors and Infection

Neoplasms located in the spine, importantly metastatic foci from distant tumors with a predilection for lumbosacral region, can change the anatomical structure of vertebral bodies, thereby hinder identification of the target level on imaging [6,26]. Similarly, vertebral infections (e.g., osteomyelitis/diskitis) result in the destruction of the vertebral bodies. Chronic infections can cause the vertebral bodies to fuse; therefore, meticulous evaluation of preoperative imaging should be performed to avoid surgical errors [6,26].

Previous Spine Surgery

Surgeries on the lumbosacral region, including complex procedures that involve partial or complete resection of parts of the vertebral column, can alter the typical anatomical features and common landmarks in lumbosacral spine. This deformation complicates anatomic localization of the target vertebrae in repeat lumbosacral surgery. In addition, bony defects and fractures can make localization on x-rays more challenging. Moreover, scar tissue makes it more difficult to identify the correct level intraoperatively. Some patients may have prior instrumentation, including screws, rods, and cages that can interfere with localization; therefore, the surgeon should thoroughly review the surgical history of the patient to minimize the risk of performing surgery at the wrong level [6,26].

Obesity and Osteoporosis

Many patients undergoing spine surgery have comorbid conditions, such as obesity and osteoporosis. In the light of the increasing incidence of obesity, the number of spinal surgeries performed on this population will also continue to rise. [32]. Elderly patients are undergoing spine procedures more frequently along with the increasing incidence of osteoporosis in these individuals [33]. Osteoporosis can decrease the disc height and collapse the vertebral bodies, and give them a wedge-shaped, flat, or biconcave shape [29]. These deformities alter the spine’s typical radiologic appearance and can complicate spinal localization. The collapsed biconcave vertebrae have a classic radiologic presentation and are referred to as “fish vertebrae” [29]. Furthermore, obese patients with excess visceral fat (obscuring the lumbosacral region) and those with decreased bone mineral density may lead to the inability to visualize the correct operative level [1,34]. Therefore, it is critical to obtain a high-quality x-ray to count the levels reliably [35].

Strategies to Prevent Surgery at the Wrong Level

Given these lumbosacral vertebral anomalies and patient-related factors that can increase the risk of lumbosacral surgery at the wrong level, it is essential to follow a systematic approach to ensure targeting the correct level(s). This is initially established in the doctors where the patient’s consent for the correct side and level(s) is obtained [34]. In addition, evaluation of preoperative imaging to localize potential anatomical variants should be performed. In cases where preoperative imaging is suboptimal, repeat x-rays should be taken [5]. Fiducial markers placed preoperatively by an interventional radiologist may be necessary in patients with expected enumeration difficulty [34,36,37]. The surgeon should have the imaging available to review in the OR. Intra-operative x-rays must be of good quality in order to localize the target level(s) appropriately [34]. Assistance from radiology can also be helpful if the intraoperative images are unclear. To facilitate guidance to the intervertebral disc of interest, the surgeon may place a spine needle between spinous processes; and then perform a lateral x-ray [38]. Ladak et al. reported a protocol in which a second time-out is conducted so that all members of the surgical team confirm the level marked on the image before continuing with the procedure [39]. The primary surgeon also should mark the surgery site personally. In the setting of complex anatomy, several intraoperative x-ray images should be taken to confirm the correct surgical side and level(s). In cases with instrumentation, an additional intraoperative x-ray is preferable prior to wound closure to confirm successful instrumentation and verify the levels [34]. Intraoperative CT, spinal neuronavigation, and transligamentous ultrasound may be also utilized to assist in the proper identification of the target level(s), minimizing the risk of lumbar spine surgery at the wrong level [1,3].

## Conclusions

Surgery in the lumbosacral spine at the wrong level can be a very problematic ordeal for surgeons and their patients, and typically results in litigation. It can lead to unnecessary surgeries and additional risks for the patient with subsequent damage to the relationship between surgeon and patient. Several factors can increase the risk of such surgeries. In this literature review, we described several lumbosacral anatomical variations and patient characteristics that can lead to surgery at the wrong level, and have emphasized the importance of the preoperative and intraoperative imaging in identification of these variants. Significantly, working with radiologists enables the surgeon to identify potential risk factors and clarify imaging.
